# Literary tastes are as heritable as other human phenotypes: Evidence from twins’ library borrowing

**DOI:** 10.1371/journal.pone.0306546

**Published:** 2024-07-05

**Authors:** Mads M. Jæger, Stine Møllegaard, Ea H. Blaabæk

**Affiliations:** 1 Department of Sociology, University of Copenhagen, Copenhagen, Denmark; 2 The Danish Evaluation Institute, Copenhagen, Denmark; 3 Statistics Denmark, Copenhagen, Denmark; Max Planck Institute for the Science of Human History, Jena, Germany, HUNGARY

## Abstract

Social science research argues that differences in individuals’ literary and cultural tastes originate in social environments. Yet, it might be that these differences are partly associated with genetic differences between individuals. To address this possibility, we use nation-scale registry data on library borrowing among Danish twins (*N* = 67,900) to assess the heritability of literary tastes. We measure literary tastes via borrowing of books of different genres (e.g., crime and biographical novels) and formats (physical, digital, and audio) and decompose the total variance in literary tastes into components attributable to shared genes (heritability), shared environments (social environment shared by siblings), and unique environments (social environments not shared by siblings). We find that genetic differences account for 45–70 percent of the total variance in literary tastes, shared environments account for almost none of the variance, and unique environments account for a moderate share. These results suggest that literary tastes are approximately as heritable as other human phenotypes (e.g., physical traits, cognition, and health). Moreover, heritability is higher for socioeconomically disadvantaged groups than for advantaged groups. Overall, our results suggest that research should consider the role of genetic differences in accounting for individual differences in literary and broader cultural tastes.

## Introduction

Do genetic differences between individuals help to explain differences in literary tastes between individuals? Theories of literary and cultural tastes in the social sciences, and empirical research that builds on these theories, provide no answer to this question, as they focus exclusively on how *social* environments (in particular, the family environment and social environments outside the family) shape literary and cultural tastes [1–4]. Yet, research outside the social sciences indicates that practically all human phenotypes are heritable to some extent, with heritability defined as the degree to which variation in a phenotype in a population is due to genetic variation between individuals in that population [5,6]. Moreover, while reading skills and the propensity to read are heritable [7], we know little about whether, conditional on reading, the taste for different literary genres and formats is also heritable. Consequently, addressing the heritability of literary tastes matters for theories of literary and cultural tastes and for research on why and what people read.

In this paper, we use nation-scale registry data on library borrowing in Denmark to assess the heritability of literary tastes. We measure differences in literary tastes via information on whether individuals borrow books from the library (i.e., whether or not they read), the genres of books they borrow (e.g., fiction vs. non-fiction), and their preferred book formats (physical books vs. E-books and E-audio books) [8,9]. Our data, which include all books in all public libraries (*N* = 9,165,031 books), effectively cover the entire universe of library borrowing in Denmark.

We use data on non-deceased, adult twins, and statistical methods from behavioral genetics (the ACE model), to decompose the total variance in literary tastes into variance components attributable to shared genes (heritability), shared environments (social environments shared by siblings from the same family, e.g., the rearing environment and the neighborhood), and unique environments (social environments not shared by siblings, e.g., friends and jobs) [10]. Our data differ from those used in existing research because they include all Danish twins (rather than a sample) and include information on literary behavior (the books individuals borrow from the library) rather than self-reports (the books individuals say they read). While library borrowing does not capture individuals’ literary tastes in their entirety, we link the registry data on library borrowing with survey data to show that library borrowing correlates with individuals’ self-reported literary tastes and engagement in broader cultural activities (e.g., attendance at stage art and museums; see S1 and S2 Tables in S1 File). Consequently, library borrowing is a valid proxy for literary tastes, which are a key ingredient in broader cultural tastes [8,9]. Moreover, although we might expect wealthier individuals not to use libraries and instead buy books, this is not the case in Denmark in which individuals with very high income or wealth are about as likely to borrow books from the library as are individuals with very low income or wealth (S1 Fig in S1 File). We make four contributions to existing research.

First, we provide baseline empirical estimates of the heritability of literary tastes. To our knowledge, no research has estimated the heritability of literary tastes. Evidence from research outside the social sciences suggests that traits that resemble or reflect literary and cultural tastes are moderately heritable. For example, the heritability of reading [7], listening to music [11], attitudes towards music and arts [12,13], tastes in food [14,15], and aesthetic appraisals [16] lies in the range 0.20–0.50. We add by addressing literary tastes, a key ingredient in broader cultural tastes.

Second, we use metadata on each library book, e.g., genre (fiction vs. non-fiction, crime novel vs. biography etc.) and format (physical book vs. E-book, text vs. audio), to estimate the heritability of different literary genres and formats. By doing so, we assess if, conditional on borrowing books from the library (i.e., reading in the first place), heritability is similar across genres with different complexity, e.g., crime novel (“easy”) vs. developmental novel/*Bildungsroman* (“hard”) [8,9], and different ways of consuming literature (e.g., physical books vs. E-books). As reading physical and digital books, or listening to audiobooks, requires different skills and resources (e.g., cognitive skills, access to a physical library, or a reader-friendly tablet), we argue that genetic and environmental variation might differ across formats as well as across genres.

Third, we explore heterogeneity in the heritability of literary tastes. We address demographic heterogeneity by estimating heritability separately for male and female twins and separately for twins born in ten-year birth cohorts between 1951 and 2002. We motivate these analyses from evidence that women read more than men [8,17], even within families [18], and evidence that heritability varies across the life course [19]. We address socioeconomic heterogeneity by estimating heritability separately for individuals with high and low education and with high and low income and consider two scenarios. In the first scenario, heritability is higher for individuals with high education and income (i.e., the privileged) than for individuals with low education and income (i.e., the less privileged). We motivate this scenario from evidence that, due to being raised in higher-quality rearing environments, the privileged are better able to realize their genetic potential for reading than the less privileged [20]. If true, this scenario predicts higher heritability among the privileged. In the second scenario, heritability is lower for the privileged than for the less privileged. We motivate this scenario from evidence that the privileged have more “omnivorous” cultural tastes than the less privileged [9], which leads them to engage in diverse literary genres and formats, including genres and formats that do not necessarily match their genetic predispositions. If true, this scenario predicts lower heritability among the privileged.

Fourth, we focus on a best-case context in which we would expect heritability of literary tastes to be high. Denmark has high income redistribution, a generous social security system, and free public services, including libraries [21]. Research shows that heritability is higher in more egalitarian contexts than in less egalitarian ones [22,23], arguably because more egalitarian contexts suppress inequality in shared environments that, in turn, lead to a larger relative role of genetic variation [24]. This means that our heritability estimates for Denmark are likely upper-bound estimates.

Our empirical results are relevant beyond research on literary and cultural tastes. For one, research in the social sciences argues that high-status cultural tastes (e.g., a taste for highbrow literary genres such as *Bildungsroman*) signal competence, which in turn yields unfair economic and social advantage [1]. In support of this argument, research finds positive associations between high-status cultural tastes and income [25], social and occupational status [26], network quality [27], and elite position [28]. Research also finds that parents transmit high-status cultural tastes to their children [29], which in turn are positively associated with children’s educational [30] and socioeconomic success [31,32]. All of this research interprets cultural tastes, their intergenerational transmission, and their association with economic and social advantage, as originating in social environments. Our research challenges this interpretation by showing that genetic differences account for a non-trivial share of individual differences in literary tastes, a finding that theories of literary and cultural tastes should consider.

## Materials and methods

### Data

We analyze nation-scale registry data with information on the daily status of all materials in all public libraries in Denmark in 2021. We focus on library books from the adult book collection (*N* = 9,165,031 books) and do not include children’s books, music, games, newspapers etc. We construct 19 binary indicators (which take the value zero if an individual borrowed no books of a particular genre/format in 2021 and the value 1 if she borrowed at least one book) to capture the taste for different literary genres and formats. We derive genres and formats from the official categories in the library system that librarians use to search for books. Our indicators include: (a) borrowing any book from the adult book collection in 2021; (b) borrowing any book belonging to each of the five most popular fiction genres (crime, biographical novel, thriller, historical novel, family novel); (c) borrowing any book belonging to each of the five most popular non-fiction genres (biography, home, medicine, education, English literature); (d) borrowing any book belonging to each of three highbrow genres (experimental novel, developmental novel [*Bildungsroman*], and description of societies); (e) borrowing any fiction book or any non-fiction book; and (f) borrowing books belonging to each of three different formats (physical book, E-book, and E-audiobook). Borrowing E-books and E-audiobooks is done via the free app E-reolen (“E-shelf”), developed and supported by a non-profit organization run by public libraries.

We use unique personal identifiers in the Danish registry data (akin to social security numbers) to link information on individuals’ library borrowing in 2021 with registry data on their socioeconomic and demographic characteristics (e.g., sex, age, education, and income), also measured in 2021. Our analytical sample consist of all non-deceased twins born 1951–2002 who resided in Denmark on 31 December 2020. This restriction ensures that (a) all twins in the sample are at least 18 years old when we measure their library use and (b) we can identify the mothers of almost all twins in these birth cohorts (information becomes increasingly sparse as we move further back in time).

### Methods

We estimate the ACE model as a Structural Equation Model using LAVAAN in *R*. We adopt a version of the ACE model that takes into account that we use binary (0–1) indicators of literary tastes. As the registry data do not include information on zygosity, we rely on a methodological approach that uses twins’ sex to estimate heritability [33–38]. This approach builds on three arguments. First, as opposite-sex twins (male and female) can only be dizygotic (DZ), the population of opposite-sex twins is comprised solely of DZ twins assumed to share on average 50% of segregating genes (*N* = 11,619 twin pairs). Second, the population of same-sex twins comprises a mix of monozygotic (MZ) twins, assumed to share 100% of segregating genes, and DZ twins, assumed to share on average 50% of segregating genes (*N* = 22,331 twin pairs). If we assume an equal distribution of men and women among DZ twins, we can approximate the proportion of MZ twins in the population of same-sex twins by means of Weinberg’s rule [38,39]. Third, to account for the mix of MZ and DZ twins in the population of same-sex twins, we adjust the assumed genetic correlation in the population of same-sex twins using Weinberg’s rule (N_Same-sex_ -N_Opposite-sex_)/N_Same-sex_ + 0.5 * (N_Opposite-sex_/N_Same-sex_) [38,39]. In our data, we estimate this genetic correlation to be 0.74. In S3 Table in S1 File, we report polychoric correlations for same-sex and opposite-sex twins (we report polychoric correlations because our indicators of literary tastes are binary). To estimate heritability separately for male and female twins, we use the approach proposed by [36] that substitutes opposite-sex DZ twins with same-sex closely spaced (non-twin) siblings (*N* = 166,897 sibling pairs). In S4 Table in S1 File, we show that although ACE models that use this approach return smaller estimates of C, our main results remain robust. Moreover, S5a, S5b and S6 Tables in S1 File summarize results from ACE models separately by genre, format, age, and sex. Finally, as a supplement to the ACE model, we also estimate ADE models that take into account that genetic variation in literary tastes might also be due to non-additive genetic influences [40]. S7 Table in S1 File summarizes results from ADE models. We discuss assumptions and limitations in the ACE model in S1 File.

## Results

We begin by briefly describing our data and patterns of library borrowing in Denmark. After this, we present standardized estimates of the heritability of different literary genres and formats. To this end, we use the ACE model that we presented in the “Materials and Methods” section [10]. Finally, we present heritability estimates separately by sex, age, education, and income.

Our registry data include information on daily loans of all library books in all public libraries in Denmark in 2021. We focus on 2021, but in S8 Table in S1 File we show that including data from 2020, when libraries closed for extended periods due to the COVID-19 pandemic, does not change any of our results. We focus on books designated for, and borrowed by, adults (age 18 and older). For each book, we have metadata on type (fiction vs. non-fiction), genre (top five most popular fiction and non-fiction genres, three highbrow genres), and format (physical book, E-book, and E-audio book). Together, the top five most popular fiction and non-fiction genres account for 55 percent of all library borrowing in 2021, i.e., a considerable share. We link the registry data on library borrowing with registry data on the individuals that borrowed these books, thereby adding demographic and socioeconomic information. In 2021, 17% of the adult Danish population borrowed a least one book from the adult library collection. This share was also 17% in the population of twins that we use to estimate heritability.

**Fig 1:**
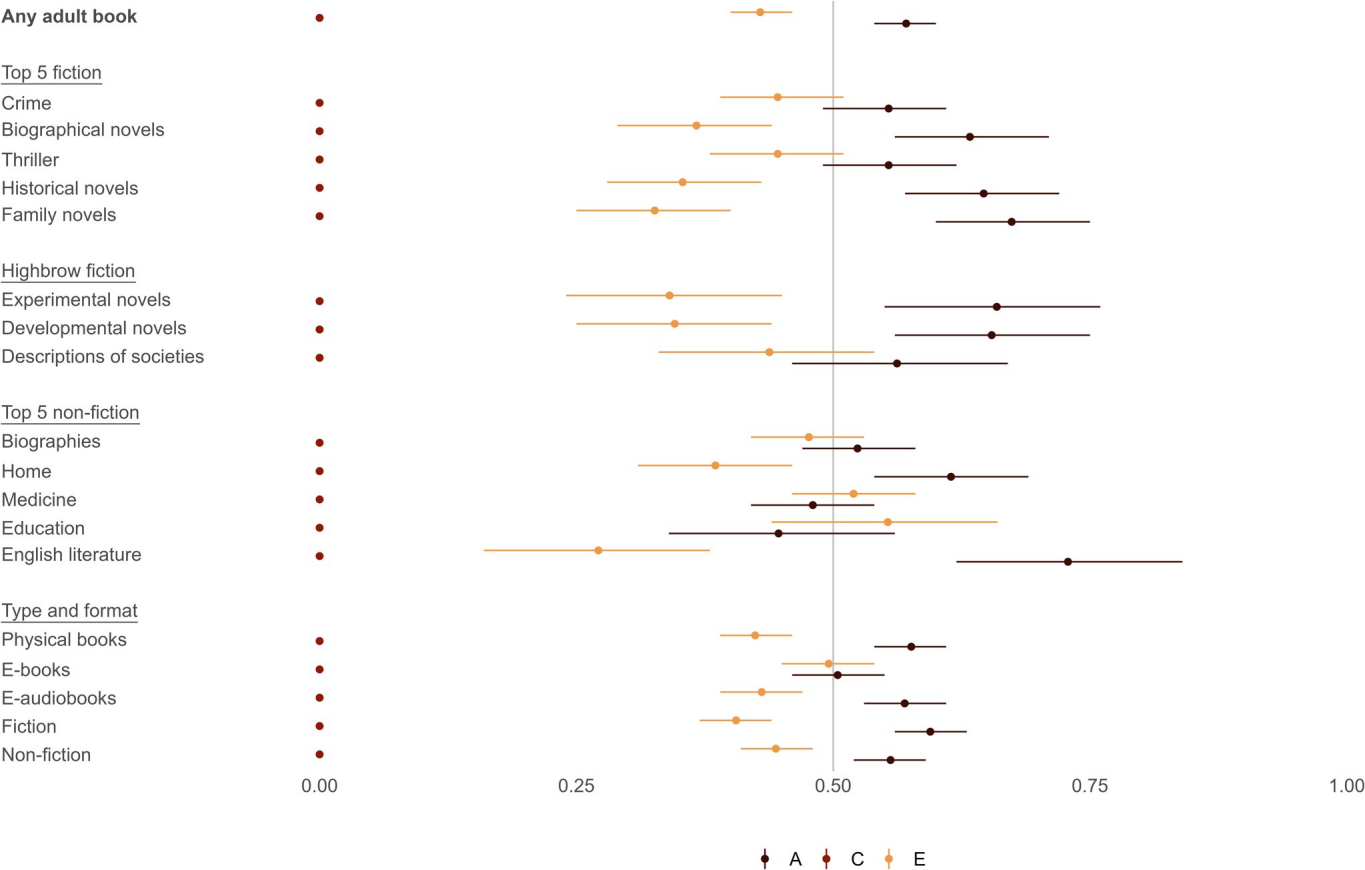
Decomposition of the total variance in borrowing of library books of different genres and formats that is attributable to shared genes (A), shared environments (C), and unique environments.

Fig 1 shows standardized heritability estimates (with 95% confidence intervals) from ACE models that decompose the total variance in the 19 binary indicators of literary tastes into components attributable to shared genes (A), shared environments (C), and unique environments (E). In the ACE model, the A component captures heritability, as defined above (40) (S9 Table in S1 File provides detailed results from all ACE models). All indicators of literary taste are binary (coded 0–1) and measure if an individual borrowed at least one book of a particular genre or format in 2021.

First, we estimate the heritability of borrowing any book from the library, i.e., the extent to which individuals have a taste for reading. The estimated heritability for borrowing any book is 0.57; i.e., moderate. This estimate is comparable to that from a recent meta-study, which reports an average heritability of 0.66 for general reading [7]. Moreover, unique environments (E) account for around 43% of the total variance in borrowing any book from the library, while shared environments (C) account for none of the variance.

Second, heritability is moderate across the different literary genres and formats we consider, ranging from 0.45 (education [non-fiction]) to 0.73 (English literature [non-fiction]). Interestingly, heritability does not appear to be systematically higher or lower for any particular genre or format but, rather, moderate across genres and formats. This result suggests that although literary genres and formats differ *in kind* (e.g., in terms of popularity, complexity, and “browness”) [8,9], heritability is similar *in degree* across genres and formats–and similar to the heritability of taking out any book from the library. We must keep in mind that we analyze data from Denmark, arguably a “best case” context in which we would expect heritability to be high. Nonetheless, our heritability estimates for literary tastes for Denmark are similar to estimates for other human phenotypes. For example, the average of the 19 heritability estimates reported in Fig 1 is 0.58. This estimate is similar to the estimate of approximately 0.49 reported in a meta-analysis covering 17,804 human phenotypes [5]. In S10 Table in S1 File, we report heritability estimates for education (years of completed schooling) and income (percentile rank) using the same ACE models and population of twins that we use to estimate the heritability of literary tastes. Our heritability estimates for education and income are comparable to those reported in other research [34,41]. Consequently, our data and methods yield results that are similar to existing research when applied to other phenotypes often studied in social science research. Finally, S7 Table in S1 File summarizes results from ADE models, which capture potential non-additive genetic variance in literary tastes (the ACE model only captures additive genetic variance). These tables show that accounting for potential non-additive genetic variance does not change any of our substantive findings.

**Fig 2:**
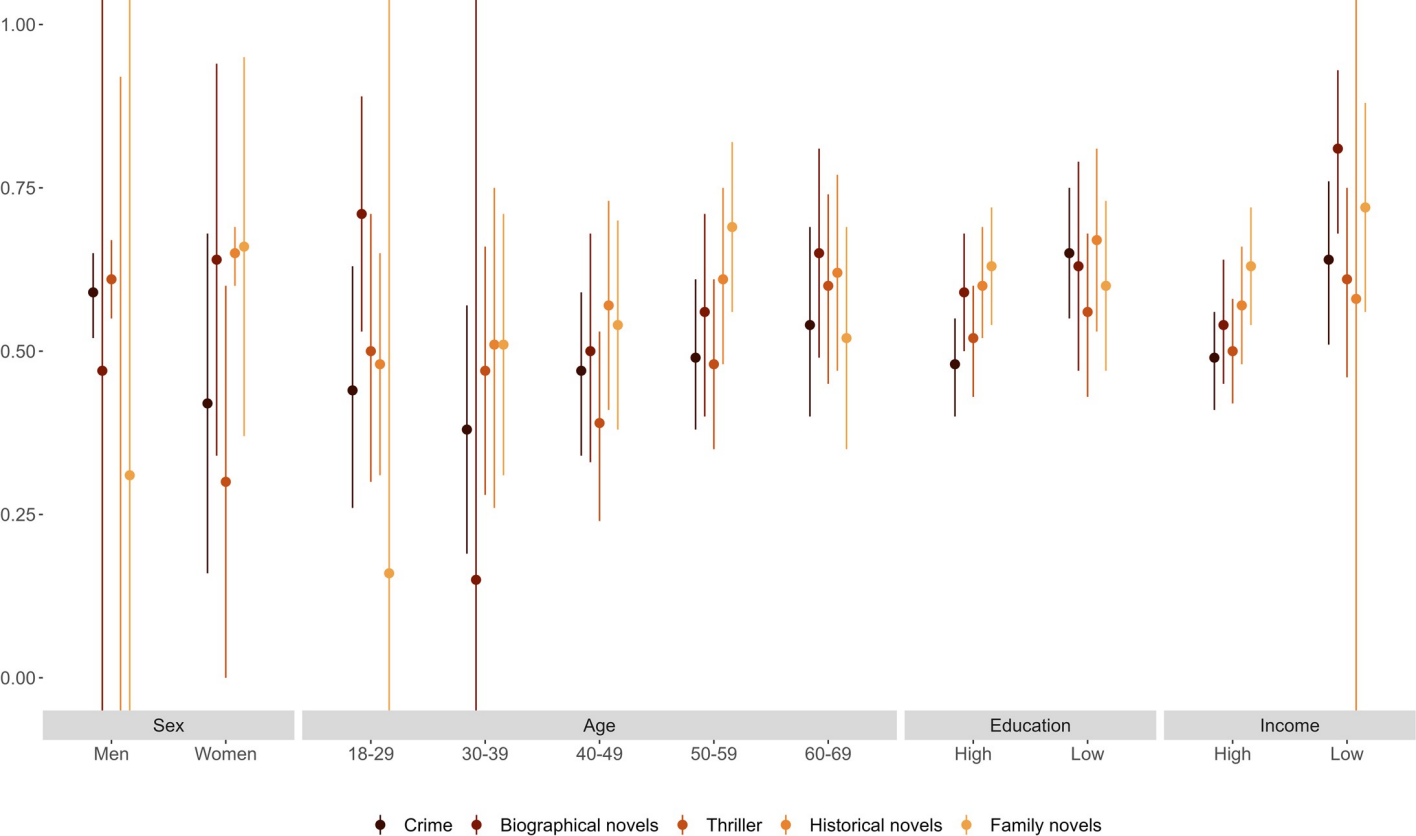
Heritability of fiction genres estimated separately by sex, age, education, and income.

**Fig 3:**
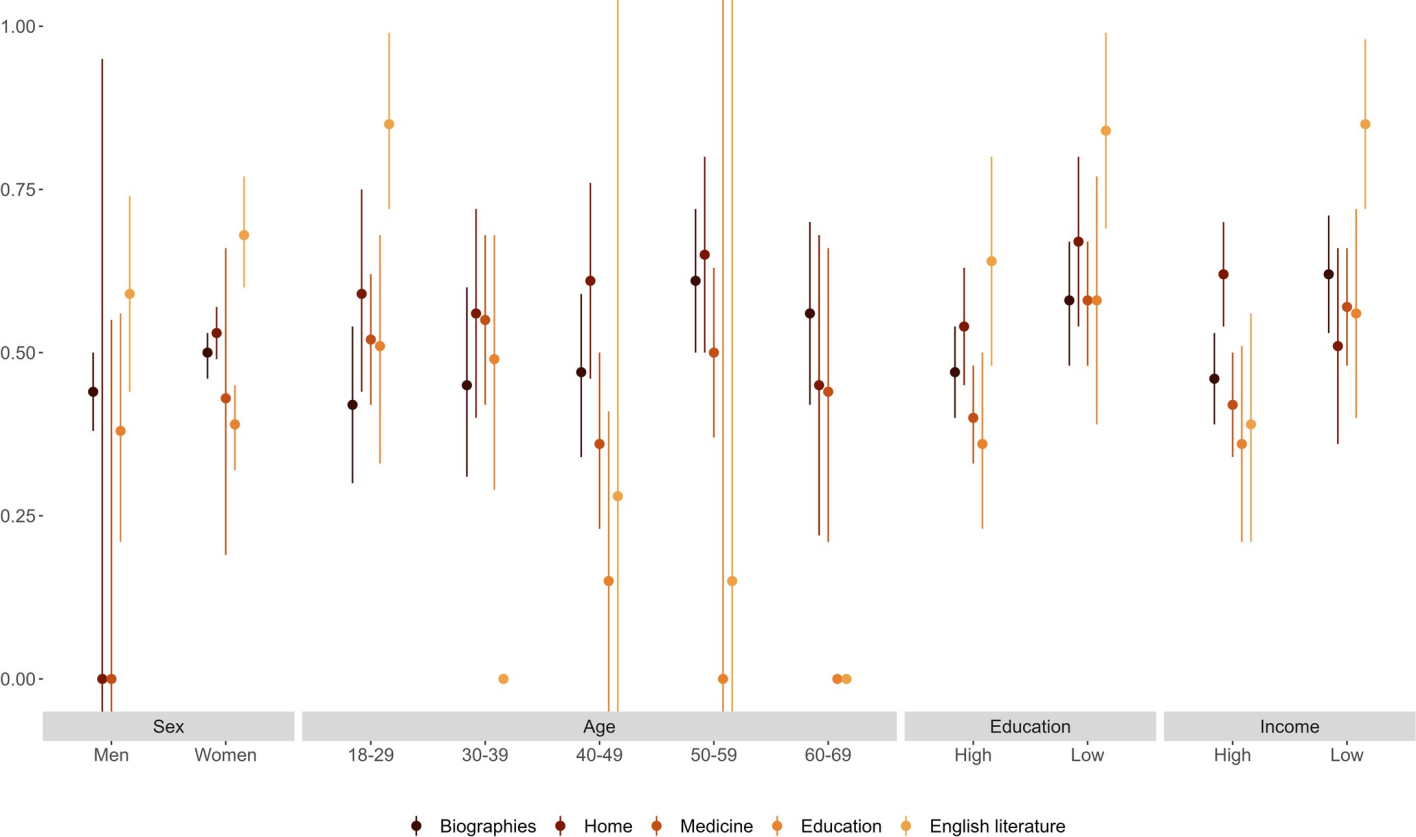
Heritability of non-fiction genres estimated separately by sex, age, education, and income.

**Fig 4:**
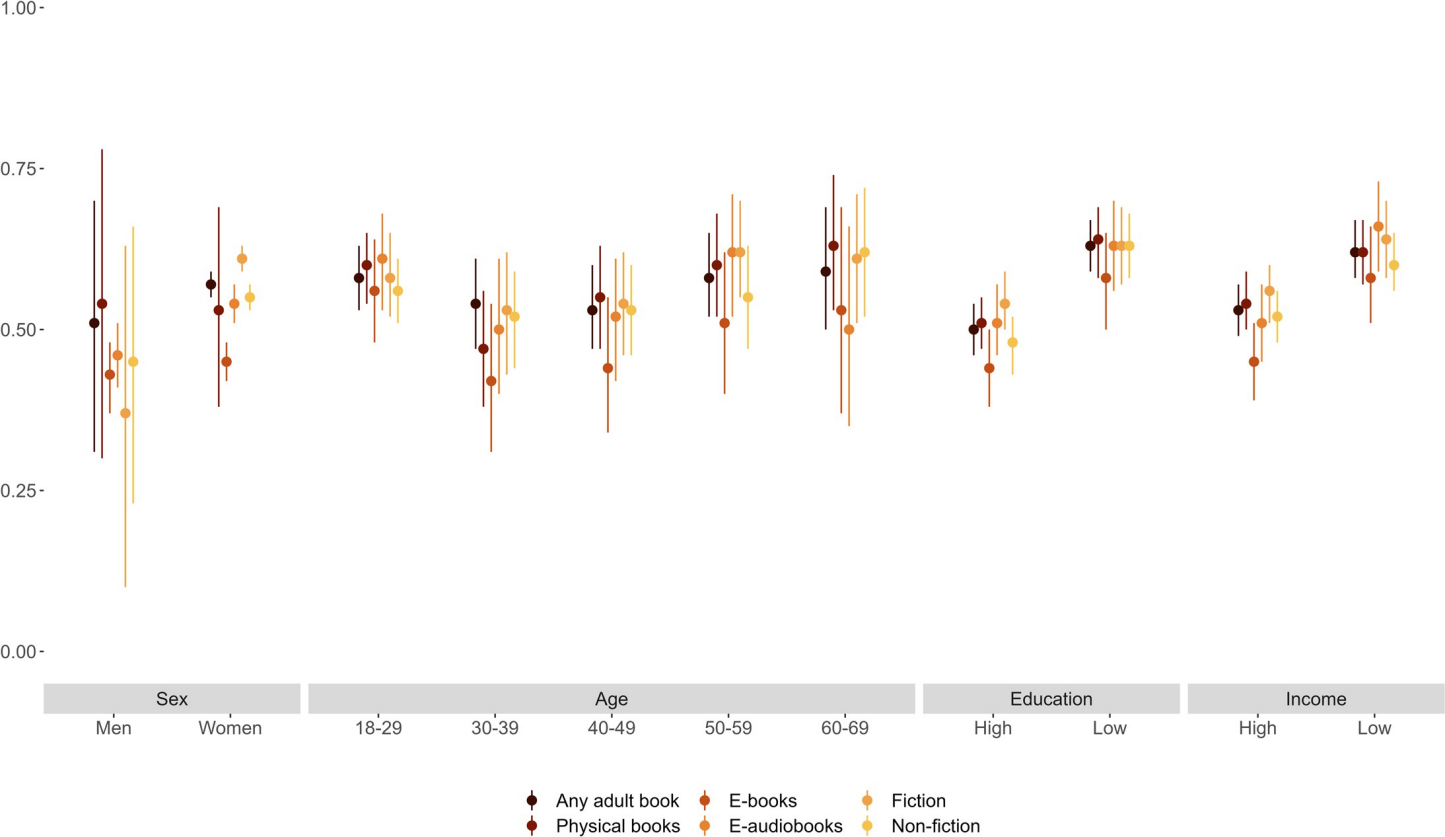
Heritability of literary formats estimated separately by sex, age, education, and income.

We now explore heterogeneity in the heritability of literary tastes. As stated above, we motivate heterogeneity by sex from evidence that women read more than men do; heterogeneity by age from evidence that heritability differs across the life course; and heterogeneity by socioeconomic position from the two scenarios described above. Figs 2–4 summarize heritability estimates (with 95% confidence intervals) by sex, age, education, and income (we measure education and income via binary variables for having completed college [vs. not] and for having a disposable income above [vs. below] the median in the sample). We provide detailed results in S11-S17 Tables in S1 File.

Figs 2–4 show that the heritability of literary tastes does not differ appreciably between men and women (in most cases, differences in the estimated heritability are not statistically significant across literary genres and formats). While heritability estimates are mostly similar between men and women, the large confidence intervals associated with these estimates (particularly for genres that men prefer less often than women, such as biographical novels) mean that, even with population data, results could mask sex differences in the heritability of literary tastes. The genres that stand out with lower heritability are genres less often borrowed, leading to more noise, captured by E. While the heritability of most fiction genres appears to increase with age (Fig 2) and to vary across formats (Fig 4), in most cases these differences are not statistically significant. Moreover, the heritability of non-fiction genres also does not vary with age (Fig 3). Overall, our results suggest that the heritability is similar by sex and age.

With regard to socioeconomic heterogeneity, Figs 3 and 4 show that, for most non-fiction genres and formats, heritability is higher for individuals with low education (less than 15 years of education) or low income (below-median disposable income) than for individuals with high education (15 years or more) or high income (above-median disposable income). We observe a similar pattern for fiction genres, although differences between heritability estimates often do not reach statistical significance at *p* < 0.05 (S14a and S15a Tables in S1 File summarize results from *Z* tests).

## Discussion

In this paper, we assess the heritability of literary tastes. This matters because social science research interprets literary and broader cultural tastes, their intergenerational transmission, and their association with economic and social inequality as originating in social environments, not genetic differences between individuals [1,18,29]. Moreover, while we know that the propensity to read is heritable [7], we know little about the heritability of the taste for different literary genres and formats. We analyze nation-scale registry data with Danish twins, include multiple dimensions of literary tastes, and measure behavior rather than self-reports. Our main result is that literary tastes are moderately heritable, approximately to the same extent as most other human phenotypes (e.g., physical traits, cognition, and health). Furthermore, social environments not shared by twins account for considerably more of the variance in literary tastes than social environments shared by twins. This result suggests that social environments outside, but not inside, the family account for individual differences in book borrowing and literary tastes [2].

Our results speak to social science research in several ways. First, they suggest that theories of literary and cultural tastes should incorporate genetic variation between individuals, in addition to environmental variation, when theorizing cultural tastes and how they operate. We note that, as with any ACE design, our results *describe* the extent to which different variance components account for the total variance in library borrowing and literary tastes. Importantly, the ACE model does not *explain* how (or why) genetic and environmental differences lead to differences in literary tastes in a population. Accordingly, our result that shared environments do not account for much of the variance does not mean that parents cannot affect children’s literary tastes. For example, shared environments not accounting for much of the variance in literary tastes might be due to the generous social security system in Denmark, which suppresses variance in shared environments in the population. Furthermore, gene-environment interactions, like parents reinforcing children’s genetic predispositions, would be captured by the genetic component and would not negate the role of parenting [42,43]. With regard to reading practices, our results relate to conflicting evidence that, on the one hand, shared environments account for a non-trivial share of the variance in the time children spend reading [11] and, on the other hand, evidence that these shared environments have little impact on adults’ reading practices [44]. This discrepancy might suggest that heritability, and the relative importance of shared environments, varies over the life course. Moreover, research increasingly uncovers how heritability varies across cultural and social contexts [22,24,36]. Accordingly, we encourage research that addresses heterogeneity in the heritability of literary and cultural tastes.

Second, while our results suggest that genetic differences accounts for a non-trivial share of individual differences in literary tastes, they are uninformative about how genetic differences impact tastes or the extent to which policy might affect tastes. We encourage research that unpacks whether the heritability of literary and cultural tastes links to differences in cognitive skills or personality. Extant research shows that personality traits, like openness to experience and conscientiousness, shape cultural and literary preferences [45,46]. As personality likely precedes adult literary tastes, it might be that personality mediates some of the genetic influence on literary tastes. Furthermore, the finding that a phenotype like literary tastes is heritable does not mean that policy cannot affect (inequality in) literary tastes, as demonstrated by the fact that policy can redress inequality in highly heritable phenotypes like eyesight and diabetes by means of glasses and insulin [42,47]. Genetic influences are inherently complex and operate via environments and phenotypes [3,4]. This means that other heritable phenotypes, for example, cognitive skills and personality, as well as self-selection into social environments based on genotype, in part explain why genetic differences link to individual differences in literary tastes. Accordingly, we encourage research that unpacks how social environments and genetic predispositions interact to shape variation in literary tastes. For example, genetic differences might explain differences in literary taste because parents reinforce children’s genetic predispositions. In line with this idea, research shows that parents offer activities and rearing environments that match children’s genetic predispositions [43]. Another interesting avenue for research would be to explore the extent to which adults self-selecting into social environments based on genetic predispositions explain our empirical results.

Finally, while we find little evidence of heterogeneity by sex and age, we find that the heritability of book borrowing and literary tastes is higher among the less privileged than among the privileged. While differences in heritability are not large, they are consistent with the idea that, due to having more omnivorous tastes, the privileged (i.e., those with high education or income) are more likely than the less privileged (i.e., those with low education or income) to have a taste for diverse literary genres and formats, including genres and formats that do not necessarily match their genetic predispositions. We back this interpretation by research suggesting that the privileged are more attuned to external status cues (e.g., from media or peers) than the less privileged [48], meaning that they more often adopt tastes regarded as “hip” and that have high status, such as omnivorous tastes. Moreover, the privileged might have more omnivorous tastes than the less privileged due to exposure to diverse tastes in the family of origin or among their peers [2,9,44]. The Danish context likely also plays a role. The high level of income redistribution in Denmark means that, compared to the United States and elsewhere, low inequality makes it possible for the less privileged to provide a rearing environment of similar quality to the one provided by the privileged. This (more) equal playing field means that children in less privileged families might have the same potential to realize their genetic potential as children in privileged families, which manifests in higher (relative) heritability [49,50]. Finally, if heritable phenotypes like personality and cognition mediate our results, then variation in these phenotypes across socioeconomic groups (and variation in the extent to which social environments affect personality and cognition) might explain why heritability in literary tastes is higher among the less privileged than among the privileged.

## Supporting information

S1 File We provide supporting tables and figures in the S1 File. (PDF)
