# Supplementary material for: Literary tastes are as heritable as other human phenotypes: Evidence from twins’ library borrowing
Source: PLoS One. 2024 Jul 5;19(7):e0306546. doi: 10.1371/journal.pone.0306546 (PMC11226123; doi:10.1371/journal.pone.0306546)
Supplement: S1 File — (PDF) [file pone.0306546.s001.pdf]

Supporting Information for

Literary tastes are as heritable as other human phenotypes: Evidence from twins' library borrowing

Mads M. Jæger, Stine Møllegaard, and Ea H. Blaabæk

Corresponding author: Mads Meier Jæger

Email: mmj@soc.ku.dk

This PDF file includes:

Discussion of assumptions and limitations in the ACE model

Tables S1 to S17

Figure S1

Assumptions and Limitations in the ACE Model

The first assumption in the ACE model, the Equal Environments Assumption (EEA), is that MZ and DZ twins share rearing environments to the same extent. Some research argues that, compared to DZ twins, MZ twins are more often in contact and treated more similarly by parents. If true, this would violate the EEA, leading to greater (non-genetic) similarity between MZ twins and thus to inflated estimates of A. Research that addresses the EEA finds it to hold in most cases and, when violated, leads to moderate bias [1,2]. Violation of the EEA creates further bias if parents treat same-sex twins more similarly than opposite-sex DZ twins because of sex stereotypes. While this assumption might not hold in the main analyses, comparing same-sex twins with same-sex closely spaced siblings separately by sex yields similar results (S1_File S4).

The second assumption in the ACE model is that there is no assortative mating based on genotype, i.e., (future) parents not selecting into couples based on genetic similarities. If parents are more genetically similar than random individuals, DZ twins share more than 50% of segregating genes, as assumed in the ACE model. In that case, the ACE model overestimates the role of shared environments (C) [3,4]. It is possible to test the implications of this assumption by varying twins' assumed genetic correlation when estimating the ACE model. However, as our estimates of the role of shared environments are consistently (close to) zero, it is unlikely that a violation of this assumption affects our results.

The third assumption in the ACE model is that genetic effects are additive, i.e., there is no genetic dominance and no gene-gene interactions. Genetic dominance and gene-gene interactions might manifest in rMZ, i.e., correlations between MZ twins' literary tastes, being more than twice that of rDZ, i.e., correlations between DZ twins' literary tastes. We can address this assumption based on model fit statistics. S1_File S3 shows that, in many cases, the intra-class correlation among same-sex twins is more than twice that of the intra-class correlation of opposite-sex twins. However, the intra-class correlation for same-sex twins is not twice that of same-sex closely spaced siblings, and results are robust to using closely spaced siblings as substitutes for DZ twins (see S1_File S4 and S5a-S5b). Finally, S1_File S7 summarizes results from ADE models that, unlike the ACE model, incorporate non-additive, genetic dominance as well as additive genetic influences [5]. Results from ADE models lead to the same substantive conclusions as the ones we present in the main analysis.

The fourth assumption in the ACE model is that there are no gene-environment interactions (GxE). GxE implies that some environments are more conducive to the expression of genetic predispositions than

others. If GxE exist, the ACE model might overestimate A [6]. We address GxE by estimating heritability separately by sex, age, income, and education and find some evidence of GxE by education and income (but not by sex and age). We discuss these findings in the main text.

References used in Assumptions and Limitations in the ACE Model

1. D. Conley, E. Rauscher, C. Dawes, P. K. E. Magnusson, M. L. Siegal. Heritability and the Equal Environments Assumption: Evidence from Multiple Samples of Misclassified Twins. Behavior Genetics. 2013; 43: 1-12.
2. J. Felson. What can we learn from twin studies? A comprehensive evaluation of the equal environments assumption. Social Science Research. 2014; 43: 184-199.
3. S. Scarr-Salapatek. Race, social class, and IQ. Science. 1971; 174: 1285-1295.
4. D. N. Figlio, J. Freese, K. Karbownik, J. Roth. Socioeconomic status and genetic influences on cognitive development. Proceedings of the National Academy of Sciences. 2017; 114: 13441-13446.
5. C. V. Dolan, D. I. Boomsma, M. G. Nivard, M. C. Neale. A Note on Jöreskog’s ACDE Twin Model: A Solution, Not the Solution. Structural Equation Modeling: A Multidisciplinary Journal. 2022; 29: 933-934.
6. R. Plomin, J. C. DeFries, V. S. Knopik, J. M. Niederhiser. Behavioral Genetics. New york: Worth Publishers; 2014).

Table S1. Polychoric correlations between library borrowing and self-reported cultural participation.

	Library borrowing (registry data):			
Cultural participation (survey data):	Any adult book	Physical books	E-books	E-audiobooks
Visits libraries	0.64***	0.75***	0.43***	0.36***
Reads fiction	0.46***	0.45***	0.36***	0.33***
Buys books	0.14***	0.15***	0.10***	0.08***
Goes to museum	0.25***	0.26***	0.18***	0.16***
Looks at art (museum, public, at home)	0.19***	0.20***	0.13***	0.12***
Attends “highbrow” stage art (opera, ballet, theater)	0.18***	0.19***	0.13***	0.11***
Attends “lowbrow” stage art (musical, stand-up, revue)	0.09***	0.09***	0.07***	0.05*
Goes to cinema	0.18***	0.17***	0.15***	0.13***
Attends sport event (live)	-0.05	-0.05	-0.06	-0.05
Goes to amusement park	0.06***	0.07***	0.04*	0.05**

Notes: We matched registry data on library borrowing in 2021 with survey data on cultural participation from Kulturvaneundersøgelsen (a nationally representative survey carried out by Statistics Denmark). We used the most recently available data in Kulturvaneundersøgelsen (2019-2021). All variables are dichotomous. The variables in the registry data measure if an individual borrowed a book of a particular format in 2021. The variables in the survey data measure if an individual reported participating in a cultural activity within the last three months. *N*=20,812. The table shows positive correlations between individuals’ library borrowing, self-reported reading behavior, and whether they have visited a library. It also shows positive correlations between library borrowing and participation in different cultural activities.

Table S2. Polychoric correlations between borrowing library books of different genres and self-reported literary tastes.

	Library borrowing, top 5 most popular genres (registry data):					
	Any adult book	Crime	Thriller	Family novels	Biographical novels	Historical novels
Literary tastes (survey data):						
Crime	0.26***	0.46***	0.45***	0.27***	0.17***	0.28***
Thriller and horror	0.15***	0.23***	0.25***	0.13***	0.11*	0.11*
Historical novels	0.30***	0.25***	0.29***	0.37***	0.34***	0.42***
Fantasy and science fiction	0.01	-0.03	-0.02	-0.10	-0.14	-0.06
Humor and satire	0.14***	0.08*	0.12**	0.06	0.11*	0.11**
Romance and erotica	0.23***	0.13***	0.20***	0.24***	0.14***	0.23***
Poetry	0.26***	-0.03	0.09	0.07	0.29***	0.18***

Notes: We matched registry data on library borrowing in 2021 with survey data on cultural participation from Kulturvaneundersøgelsen (a nationally representative survey carried out by Statistics Denmark). We use data from two rounds of Kulturvaneundersøgelsen that asked participants about their literary tastes (2019 Q1 and 2021 Q3). Polychoric correlations. All items are dichotomous. The variables in the registry data measure if an individual borrowed a book of a particular genre in 2021. The variables in the survey data measure if an individual reported reading literature of a particular genre (multiple genres allowed). *N*=5,680. The table shows positive correlations between the genres of books individuals borrow from the library and the genres of books they report reading.

Table S3. Intraclass polychoric correlations in (type of) library borrowing in three populations: Same-sex twins, opposite-sex twins, and same-sex closely spaced siblings.

	Same-sex twins	Opposite-sex twins	Same-sex closely spaced sibling
Any adult book	0.47	0.08	0.35
Top 5 fiction			
Crime	0.43	0.16	0.33
Biographical novels	0.49	0.14	0.36
Thriller	0.42	0.21	0.31
Historical novels	0.50	0.11	0.36
Family novels	0.52	0.004	0.39
Highbrow fiction			
Experimental novels	0.50	-0.76	0.21
Developmental novels	0.51	0.10	0.40
Descriptions of socies	0.44	0.07	0.35
Top 5 non-fiction			
Biographies	0.41	0.13	0.28
Home	0.49	0.06	0.33
Medicine	0.39	0.04	0.31
Education	0.35	0.12	0.24
English literature	0.56	0.15	0.32
Format			
Physical books	0.46	0.11	0.35
E-books	0.40	0.11	0.26
E-audiobooks	0.45	0.11	0.30
Fiction	0.48	0.09	0.36
Non-fiction	0.45	0.11	0.33

Table S4. Results from ACE models. Share of variance in (type of) library borrowing attributable to shared genes (A), shared environments (C), and unique environments (E). Models use closely spaced same-sex siblings as substitutes for dizygotic twins.

	A	A 95% conf	C	C 95% conf	E	E 95% conf	Z test (*P* value)[a]
Any adult book	0.50	[0.39-0.60]	0.10	[0.04-0.15]	0.40	[0.36-0.45]	
<u>Top 5 fiction</u>							
Crime	0.43	[0.21-0.64]	0.12	[0.00-0.23]	0.46	[0.36-0.56]	0.56
Biographical novels	0.54	[0.27-0.80]	0.10	[-0.04-0.24]	0.37	[0.24-0.49]	0.78
Thriller	0.47	[0.24-0.70]	0.08	[-0.05-0.20]	0.45	[0.34-0.57]	0.82
Historical novels	0.60	[0.35-0.84]	0.06	[-0.07-0.19]	0.34	[0.23-0.46]	0.46
Family novels	0.55	[0.30-0.81]	0.11	[-0.02-0.25]	0.34	[0.21-0.46]	0.72
<u>Highbrow fiction</u>							
Experimental novels	0.55	[0.36-0.74]	0	[0.00-0.00]	0.45	[0.26-0.64]	0.65
Developmental novels	0.46	[0.14-0.77]	0.17	[0.00-0.34]	0.38	[0.23-0.52]	0.81
Descriptions of societies	0.40	[0.05-0.75]	0.15	[-0.04-0.33]	0.45	[0.29-0.62]	0.59
<u>Top 5 non-fiction</u>							
Biographies	0.56	[0.36-0.75]	0	[-0.11-0.11]	0.44	[0.35-0.53]	0.59
Home	0.64	[0.39-0.89]	0.01	[-0.12-0.15]	0.35	[0.23-0.46]	0.30
Medicine	0.34	[0.13-0.54]	0.14	[0.03-0.25]	0.52	[0.42-0.62]	0.17
Education	0.45	[0.06-0.84]	0.02	[-0.19-0.23]	0.53	[0.35-0.72]	0.81
English literature	0.69	[0.61-0.76]	0	[0.00-0.00]	0.31	[0.24-0.39]	0.003*
<u>Type and format</u>							
Physical books	0.47	[0.35-0.59]	0.12	[0.05-0.18]	0.41	[0.36-0.47]	0.71
E-books	0.53	[0.51-0.56]	0	[0.00-0.00]	0.47	[0.44-0.49]	0.58
E-audiobooks	0.60	[0.58-0.62]	0	[0.00-0.00]	0.40	[0.38-0.42]	0.06
Fiction	0.51	[0.39-0.64]	0.10	[0.03-0.17]	0.39	[0.33-0.44]	0.90
Non-fiction	0.48	[0.37-0.60]	0.09	[0.03-0.15]	0.42	[0.37-0.48]	0.80

Notes: [a]*P* value from two-sided *Z* test. Tests whether A estimate is statistically different from A estimate for any loan of adult books. *Difference statistically significant at $P<0.05$.

Table S5a. Results from ACE models. Share of variance in (type of) library borrowing attributable to shared genes (A), shared environments (C), and unique environments (E). Separate models by sex and format and using closely spaced same-sex siblings as substitutes for dizygotic twins.

		A	A 95% conf	C	C 95% conf	E	E 95% conf	Z test (*P* value)[a]
Any adult book	Men	0.51	[0.31-0.70]	0.01	[-0.10-0.11]	0.49	[0.39-0.58]	
	Women	0.57	[0.55- 0.59]	0	[0.00- 0.00]	0.43	[0.41- 0.45]	0.55
<u>Top 5 fiction</u>								
Crime	Men	0.59	[0.52-0.65]	0	[0.00-0.00]	0.41	[0.35-0.48]	
	Women	0.42	[0.16-0.68]	0.08	[-0.06-0.22]	0.50	[0.38-0.62]	0.21
Biographical novels	Men	0.47	[-0.26-1.21]	0.01	[-0.39-0.41]	0.52	[0.17-0.87]	
	Women	0.64	[0.34- 0.94]	0	[-0.16- 0.16]	0.36	[0.22- 0.50]	0.68
Thriller	Men	0.61	[0.55-0.67]	0	[0.00-0.00]	0.39	[0.33-0.45]	
	Women	0.30	[0.00-0.60]	0.13	[-0.03-0.29]	0.57	[0.42-0.71]	0.05
Historical novels	Men	0.29	[-0.34-0.92]	0.13	[-0.20-0.47]	0.58	[0.28-0.88]	
	Women	0.65	[0.60-0.69]	0	[0.00-0.00]	0.35	[0.31-0.40]	0.26
Family novels	Men	0.31	[-0.54-1.15]	0.13	[-0.32-0.58]	0.56	[0.16-0.97]	
	Women	0.66	[0.37- 0.95]	0	[-0.16- 0.16]	0.34	[0.20- 0.47]	0.44
<u>Highbrow fiction</u>								
Experimental	Men	0	[-1.42- 1.42]	0.19	[-0.54- 0.92]	0.81	[0.11- 1.51]	
novels	Women	0.50	[0.28-0.73]	0	[0.00-0.00]	0.5	[0.27-0.72]	0.49
Developmental	Men	0.52	[-0.46-1.50]	0.04	[-0.50-0.57]	0.44	[-0.02-0.91]	
novels	Women	0.54	[0.19-0.90]	0.07	[-0.12-0.26]	0.39	[0.22-0.56]	0.97
Descriptions of	Men	0	[-1.05- 1.05]	0.21	[-0.34- 0.77]	0.78	[0.28- 1.29]	
societies	Women	0.52	[0.12-0.92]	0.06	[-0.15-0.27]	0.42	[0.23-0.61]	0.36

Notes: [a]*P* value from two-sided Z test. Tests whether A estimates are statistically different for men and women. No differences are statistically significant at $P<0.05$.

Table S5b. Results from ACE models. Share of variance in (type of) library borrowing attributable to shared genes (A), shared environments (C), and unique environments (E). Separate models by sex and format and using closely spaced same-sex siblings as substitutes for dizygotic twins.

		A	A 95% conf	C	C 95% conf	E	E 95% conf	Z test (*P* value)[a]
Top 5 non-fiction								
Biographies	Men	0.44	[0.38-0.50]	0	[0.00-0.00]	0.56	[0.50-0.62]	
	Women	0.50	[0.46-0.53]	0	[0.00-0.00]	0.50	[0.47-0.54]	0.11
Home	Men	0.00	[-0.94-0.95]	0.23	[-0.27-0.74]	0.77	[0.32-1.22]	
	Women	0.53	[0.49-0.57]	0	[0.00-0.00]	0.47	[0.43-0.51]	0.27
Medicine	Men	0.00	[-0.55- 0.55]	0.20	[-0.10- 0.50]	0.80	[0.54- 1.06]	
	Women	0.43	[0.19-0.66]	0.02	[-0.11-0.15]	0.55	[0.44-0.67]	0.16
Education	Men	0.38	[0.21- 0.56]	0	[0.00- 0.00]	0.62	[0.44- 0.79]	
	Women	0.39	[0.32-0.45]	0	[0.00-0.00]	0.61	[0.55-0.68]	0.92
English literature	Men	0.59	[0.44-0.74]	0	[0.00-0.00]	0.41	[0.26-0.56]	
	Women	0.68	[0.60-0.77]	0	[0.00-0.00]	0.32	[0.23-0.40]	0.30
Type and format								
Physical books	Men	0.54	[0.30-0.78]	0.01	[-0.12-0.14]	0.45	[0.34-0.56]	
	Women	0.53	[0.38-0.69]	0.02	[-0.06-0.10]	0.45	[0.37-0.52]	0.95
E-books	Men	0.43	[0.37-0.48]	0	[0.00-0.00]	0.57	[0.52-0.63]	
	Women	0.45	[0.42-0.48]	0	[0.00-0.00]	0.55	[0.52-0.58]	0.53
E-audiobooks	Men	0.46	[0.41-0.51]	0	[0.00-0.00]	0.54	[0.49-0.59]	
	Women	0.54	[0.51-0.57]	0	[0.00-0.00]	0.46	[0.43-0.49]	0.01*
Fiction	Men	0.37	[0.10-0.63]	0.1	[-0.05-0.24]	0.54	[0.33-0.44]	
	Women	0.61	[0.59-0.63]	0	[0.00-0.00]	0.39	[0.37-0.41]	0.08
Non-fiction	Men	0.45	[0.23-0.66]	0.04	[-0.08-0.15]	0.52	[0.37-0.48]	
	Women	0.55	[0.53- 0.57]	0	[0.00- 0.00]	0.45	[0.43- 0.47]	0.37

Notes: [a]*P* value from two-sided Z test. Tests whether A estimates are statistically different for men and women. No differences are statistically significant at *P*<0.05.

Table S6. Results from ACE models. Share of variance in borrowing any book from the library attributable to shared genes (A), shared environments (C), and unique environments (E). Separate models by sex and age group. Models use closely spaced same-sex siblings as substitutes for dizygotic twins.

	A	A 95% conf	C	C 95% conf	E	E 95% conf	Z test (*P* value)[a]
Men							
18-29	0.49	[0.42-0.55]	0	[0.00-0.00]	0.51	[0.45-0.58]	
30-39	0.38	[-0.12-0.89]	0.09	[-0.18-0.36]	0.53	[0.29-0.77]	0.67
40-49	0.49	[0.43-0.56]	0	[0.00-0.00]	0.51	[0.44-0.57]	1
50-59	0.47	[-0.01-0.96]	0.04	[-0.22-0.30]	0.49	[0.25-0.72]	0.94
60-69	0.50	[0.42-0.59]	0	[0.00-0.00]	0.50	[0.41-0.58]	0.86
Women							
18-29	0.53	[0.49-0.57]	0	[0.00-0.00]	0.47	[0.43-0.51]	
30-39	0.47	[0.14-0.81]	0.03	[-0.16-0.21]	0.50	[0.34-0.66]	0.73
40-49	0.49	[0.18-0.80]	0.02	[-0.15-0.19]	0.49	[0.34-0.64]	0.80
50-59	0.60	[0.56-0.64]	0	[0.00-0.00]	0.40	[0.36-0.44]	0.02*
60-69	0.61	[0.54-0.67]	0	[0.00-0.00]	0.39	[0.33-0.46]	0.04*

Notes: [a]*P* value from two-sided Z test. Tests whether estimates of A are statistically different when comparing the youngest age group to older age groups among men and women separately. *Difference statistically significant at $P<0.05$.

Table S7. Results from ADE models. Share of variance in (type of) library borrowing attributable to additive genes (A), non-additive genes (D), and unique environments (E), as well as total heritability (A+D). Data from 2021.

Format	A	A 95% conf	D	D 95% conf	E	E 95% conf	H (A+D)	H 95% conf
Any adult book	0	[0.00-0.00]	0.74	[0.71-0.78]	0.26	[0.22-0.29]	0.74	[0.71-0.78]
<u>Top 5 fiction</u>								
Crime	0	[0.00-0.00]	0.70	[0.63-0.78]	0.30	[0.22-0.37]	0.70	[0.63-0.78]
Biographical novels	0	[0.00-0.00]	0.80	[0.70-0.89]	0.20	[0.11-0.30]	0.80	[0.70-0.89]
Thriller	0.18	[-0.31-0.67]	0.48	[-0.14-1.10]	0.34	[0.19-0.49]	0.66	[0.51-0.81]
Historical novels	0	[0.00-0.00]	0.81	[0.72-0.90]	0.19	[0.10-0.28]	0.81	[0.72-0.90]
Family novels	0	[0.00-0.00]	0.84	[0.75-0.93]	0.16	[0.07-0.25]	0.84	[0.75-0.93]
<u>Highbrow fiction</u>								
Experimental novels	0.64	[0.13-1.15]	0.04	[-0.87-0.95]	0.32	[-0.10-0.75]	0.68	[0.25-1.10]
Developmental novels	0	[0.00-0.00]	0.82	[0.70-0.93]	0.18	[0.07-0.30]	0.82	[0.70-0.93]
Descriptions of societies	0	[0.00-0.00]	0.71	[0.58-0.84]	0.29	[0.16-0.42]	0.71	[0.58-0.84]
<u>Top 5 non-fiction</u>								
Biographies	0	[0.00-0.00]	0.67	[0.60-0.74]	0.33	[0.26-0.40]	0.67	[0.60-0.74]
Home	0	[0.00-0.00]	0.78	[0.69-0.87]	0.22	[0.13-0.31]	0.78	[0.69-0.87]
Medicine	0	[0.00-0.00]	0.62	[0.55-0.69]	0.38	[0.31-0.45]	0.62	[0.55-0.69]
Education	0.00	[0.00-0.00]	0.57	[0.43-0.71]	0.43	[0.29-0.57]	0.57	[0.43-0.71]
English literature	0	[0.00-0.00]	0.91	[0.77-1.05]	0.09	[-0.05-0.23]	0.91	[0.77-1.05]
<u>Type and format</u>								
Physical books	0	[0.00-0.00]	0.74	[0.70-0.79]	0.26	[0.21-0.30]	0.74	[0.70-0.79]
E-books	0	[0.00-0.00]	0.65	[0.59-0.71]	0.35	[0.29-0.41]	0.65	[0.59-0.71]
E-audiobooks	0	[0.00-0.00]	0.73	[0.68-0.79]	0.27	[0.21-0.32]	0.73	[0.68-0.79]
Fiction	0	[0.00-0.00]	0.77	[0.72-0.81]	0.23	[0.19-0.28]	0.77	[0.72-0.81]
Non-fiction	0	[0.00-0.00]	0.72	[0.68-0.76]	0.28	[0.24-0.32]	0.72	[0.68-0.76]

Table S8. Results from ACE models. Share of variance in (type of) library borrowing attributable to shared genes (A), shared environments (C), and unique environments (E). Pooled data from 2020 and 2021.

	A	A 95% conf	C	C 95% conf	E	E 95% conf	Z test (*P* value)[a]
Any adult book	0.62	[0.60- 0.65]	0	[0.00- 0.00]	0.38	[0.35- 0.40]	
<u>Top 5 fiction</u>							
Crime	0.63	[0.58-0.67]	0	[0.00-0.00]	0.37	[0.33-0.42]	0.93
Biographical novels	0.69	[0.63-0.75]	0	[0.00-0.00]	0.31	[0.25-0.37]	0.04*
Thriller	0.59	[0.54-0.65]	0	[0.00-0.00]	0.41	[0.35-0.46]	0.32
Historical novels	0.68	[0.62-0.74]	0	[0.00-0.00]	0.32	[0.26-0.38]	0.09
Family novels	0.71	[0.66-0.77]	0	[0.00-0.00]	0.29	[0.23-0.34]	0.004*
<u>Highbrow fiction</u>							
Experimental novels	0.48	[0.34-0.62]	0	[0.00-0.00]	0.52	[0.38-0.66]	0.05
Developmental novels	0.69	[0.61-0.76]	0	[0.00-0.00]	0.31	[0.24-0.39]	0.11
Descriptions of societies	0.61	[0.53-0.68]	0	[0.00-0.00]	0.39	[0.32-0.47]	0.70
<u>Top 5 non-fiction</u>							
Biographies	0.55	[0.51-0.59]	0	[0.00-0.00]	0.45	[0.41-0.49]	0.003*
Home	0.64	[0.58-0.69]	0	[0.00-0.00]	0.36	[0.31-0.42]	0.67
Medicine	0.52	[0.48-0.57]	0	[0.00-0.00]	0.48	[0.43-0.52]	0.0001*
Education	0.52	[0.44-0.59]	0	[0.00-0.00]	0.48	[0.41-0.56]	0.01*
English literature	0.70	[0.62-0.78]	0	[0.00-0.00]	0.30	[0.22-0.38]	0.08
<u>Type and format</u>							
Physical books	0.61	[0.58- 0.63]	0	[0.00- 0.00]	0.39	[0.37- 0.42]	0.36
E-books	0.55	[0.51-0.58]	0	[0.00-0.00]	0.45	[0.42-0.49]	0.001*
E-audiobooks	0.60	[0.56-0.64]	0	[0.00-0.00]	0.40	[0.36-0.44]	0.33
Fiction	0.63	[0.60- 0.66]	0	[0.00- 0.00]	0.37	[0.34- 0.40]	0.91
Non-fiction	0.61	[0.58- 0.64]	0	[0.00- 0.00]	0.39	[0.36- 0.42]	0.49

Notes: [a] *P* value from two-sided *Z* test. Tests whether A estimate is statistically different from A estimate for any loan of adult books. *Difference statistically significant at $P<0.05$.

Table S9. Results from ACE models. Share of variance in (type of) library borrowing attributable to shared genes (A), shared environments (C), and unique environments (E). Data from 2021.

	A	A 95% conf	C	C 95% conf	E	E 95% conf	Z test (*P* value)[a]
Any adult book	0.57	[0.54- 0.60]	0	[0.00- 0.00]	0.43	[0.40- 0.46]	
<u>Top 5 fiction</u>							
Crime	0.55	[0.49-0.61]	0	[0.00-0.00]	0.45	[0.39-0.51]	0.62
Biographical novels	0.63	[0.56-0.71]	0	[0.00-0.00]	0.37	[0.29-0.44]	0.13
Thriller	0.55	[0.49-0.62]	0	[0.00-0.00]	0.45	[0.38-0.51]	0.65
Historical novels	0.65	[0.57-0.72]	0	[0.00-0.00]	0.35	[0.28-0.43]	0.06
Family novels	0.67	[0.60-0.75]	0	[0.00-0.00]	0.33	[0.25-0.40]	0.01*
<u>Highbrow fiction</u>							
Experimental novels	0.66	[0.55-0.76]	0	[0.00-0.00]	0.34	[0.24-0.45]	0.11
Developmental novels	0.65	[0.56-0.75]	0	[0.00-0.00]	0.35	[0.25-0.44]	0.09
Descriptions of societies	0.56	[0.46-0.67]	0	[0.00-0.00]	0.44	[0.33-0.54]	0.87
<u>Top 5 non-fiction</u>							
Biographies	0.52	[0.47-0.58]	0	[0.00-0.00]	0.48	[0.42-0.53]	0.14
Home	0.61	[0.54-0.69]	0	[0.00-0.00]	0.39	[0.31-0.46]	0.27
Medicine	0.48	[0.42-0.54]	0	[0.00-0.00]	0.52	[0.46-0.58]	0.01*
Education	0.45	[0.34-0.56]	0	[0.00-0.00]	0.55	[0.44-0.66]	0.03*
English literature	0.73	[0.62-0.84]	0	[0.00-0.00]	0.27	[0.16-0.38]	0.01*
<u>Type and format</u>							
Physical books	0.58	[0.54-0.61]	0	[0.00-0.00]	0.42	[0.39-0.46]	0.83
E-books	0.50	[0.46-0.55]	0	[0.00-0.00]	0.50	[0.45-0.54]	0.02*
E-audiobooks	0.57	[0.53-0.61]	0	[0.00-0.00]	0.43	[0.39-0.47]	0.96
Fiction	0.59	[0.56-0.63]	0	[0.00-0.00]	0.41	[0.37-0.44]	0.31
Non-fiction	0.56	[0.52- 0.59]	0	[0.00- 0.00]	0.44	[0.41- 0.48]	0.48

Notes: [a] *P* value from two-sided *Z* test. Tests whether A estimate is statistically different from A estimate for any loan of adult books. *Difference statistically significant at $P<0.05$.

Table S10. Results from ACE models. Share of variance in highest completed education and individual disposable income attributable to shared genes (A), shared environments (C), and unique environments (E).

	A	A 95% conf	C	C 95% conf	E	E 95% conf
Years of completed education	0.53	[0.47-0.58]	0.24	[0.20-0.28]	0.23	[0.21-0.26]
Individual disposable income	0.58	[0.56-0.60]	0	[0.00-0.00]	0.42	[0.40-0.44]

Table S11. Results from ACE models. Share of variance in (type of) library borrowing attributable to shared genes (A), shared environments (C), and unique environments (E). Separate models by age group and fiction genre.

Top 5 fiction	Age	A	A 95% conf	C	C 95% conf	E	E 95% conf	Z test (P value)[a]
Crime	18-29	0.44	[0.26-0.63]	0	[0.00-0.00]	0.56	[0.37-0.74]	
	30-39	0.38	[0.19-0.57]	0	[0.00-0.00]	0.62	[0.43-0.81]	0.65
	40-49	0.47	[0.34-0.59]	0	[0.00-0.00]	0.53	[0.41-0.66]	0.79
	50-59	0.49	[0.38-0.61]	0	[0.00-0.00]	0.51	[0.39-0.62]	0.65
	60-69	0.54	[0.40-0.69]	0	[0.00-0.00]	0.46	[0.31-0.60]	0.39
Biographical novels	18-29	0.71	[0.53-0.89]	0	[0.00-0.00]	0.29	[0.11-0.47]	
	30-39	0.15	[-1.38-1.68]	0.26	[-0.78-1.31]	0.59	[0.07-1.11]	0.48
	40-49	0.50	[0.33-0.68]	0	[0.00-0.00]	0.50	[0.32-0.67]	0.10
	50-59	0.56	[0.40-0.71]	0	[0.00-0.00]	0.44	[0.29-0.60]	0.22
	60-69	0.65	[0.49-0.81]	0	[0.00-0.00]	0.35	[0.19-0.51]	0.62
Thriller	18-29	0.50	[0.30-0.71]	0	[0.00-0.00]	0.50	[0.29-0.70]	
	30-39	0.47	[0.28-0.66]	0	[0.00-0.00]	0.53	[0.34-0.72]	0.83
	40-49	0.39	[0.24-0.53]	0	[0.00-0.00]	0.61	[0.47-0.76]	0.39
	50-59	0.48	[0.35-0.61]	0	[0.00-0.00]	0.52	[0.39-0.65]	0.87
	60-69	0.60	[0.45-0.74]	0	[0.00-0.00]	0.40	[0.26-0.55]	0.44
Historical novels	18-29	0.48	[0.31-0.65]	0	[0.00-0.00]	0.52	[0.35-0.69]	
	30-39	0.51	[0.26-0.75]	0	[0.00-0.00]	0.49	[0.25-0.74]	0.84
	40-49	0.57	[0.41-0.73]	0	[0.00-0.00]	0.43	[0.27-0.59]	0.45
	50-59	0.61	[0.48-0.75]	0	[0.00-0.00]	0.39	[0.25-0.52]	0.24
	60-69	0.62	[0.47-0.77]	0	[0.00-0.00]	0.38	[0.23-0.53]	0.22
Family novels	18-29	0.16	[-0.81-1.14]	0.31	[-0.20-0.82]	0.52	[0.05-0.99]	
	30-39	0.51	[0.31-0.71]	0	[0.00-0.00]	0.49	[0.29-0.69]	0.49
	40-49	0.54	[0.38-0.70]	0	[0.00-0.00]	0.46	[0.30-0.62]	0.45
	50-59	0.69	[0.56-0.82]	0	[0.00-0.00]	0.31	[0.18-0.44]	0.29
	60-69	0.52	[0.35-0.69]	0	[0.00-0.00]	0.48	[0.31-0.65]	0.47

Notes: [a]P value from two-sided Z test. Tests whether estimates of A are statistically different when comparing the youngest age group to older age groups. *Difference statistically significant at $P<0.05$.

Table S12. Results from ACE models. Share of variance in (type of) library borrowing attributable to shared genes (A), shared environments (C), and unique environments (E). Separate models by age group and non-fiction genre.

Top 5 non-fiction	Age	A	A 95% conf	C	C 95% conf	E	E 95% conf	Z test (*P* value)[a]
Biographies	18-29	0.42	[0.30-0.54]	0	[0.00-0.00]	0.58	[0.46-0.70]	
	30-39	0.45	[0.31-0.60]	0	[0.00-0.00]	0.55	[0.40-0.69]	0.76
	40-49	0.47	[0.34-0.59]	0	[0.00-0.00]	0.53	[0.41-0.66]	0.57
	50-59	0.61	[0.50-0.72]	0	[0.00-0.00]	0.39	[0.28-0.50]	0.02*
	60-69	0.56	[0.42-0.70]	0	[0.00-0.00]	0.44	[0.30-0.58]	0.14
Home	18-29	0.59	[0.44-0.75]	0	[0.00-0.00]	0.41	[0.25-0.56]	
	30-39	0.56	[0.40-0.72]	0	[0.00-0.00]	0.44	[0.28-0.60]	0.79
	40-49	0.61	[0.46-0.76]	0	[0.00-0.00]	0.39	[0.24-0.54]	0.85
	50-59	0.65	[0.50-0.80]	0	[0.00-0.00]	0.35	[0.20-0.50]	0.58
	60-69	0.45	[0.22-0.68]	0	[0.00-0.00]	0.55	[0.32-0.78]	0.32
Medicine	18-29	0.52	[0.42-0.62]	0	[0.00-0.00]	0.48	[0.38-0.58]	
	30-39	0.55	[0.42-0.68]	0	[0.00-0.00]	0.45	[0.32-0.58]	0.72
	40-49	0.36	[0.23-0.50]	0	[0.00-0.00]	0.64	[0.50-0.77]	0.06
	50-59	0.50	[0.37-0.63]	0	[0.00-0.00]	0.50	[0.37-0.63]	0.81
	60-69	0.44	[0.21-0.66]	0	[0.00-0.00]	0.56	[0.34-0.79]	0.52
Education	18-29	0.51	[0.33-0.68]	0	[0.00-0.00]	0.49	[0.32-0.67]	
	30-39	0.49	[0.29-0.68]	0	[0.00-0.00]	0.51	[0.32-0.71]	0.88
	40-49	0.15	[-0.10- 0.41]	0	[0.00- 0.00]	0.85	[0.59- 1.10]	0.02*
	50-59	0	[-2.15- 2.15]	0.23	[-1.15- 1.61]	0.77	[-0.07- 1.61]	0.64
	60-69	0	[0.00-0.00]	0.39	[0.33-0.46]	0.61	[0.54-0.67]	0
English literature	18-29	0.85	[0.72-0.99]	0	[0.00-0.00]	0.15	[0.01-0.28]	
	30-39	0	[0.00-0.00]	0.53	[0.34-0.73]	0.47	[0.27-0.66]	0
	40-49	0.28	[-1.01-1.58]	0.10	[-0.62-0.81]	0.62	[0.02-1.22]	0.39
	50-59	0.15	[-1.43-1.73]	0.28	[-0.54-1.10]	0.57	[-0.20-1.34]	0.39
	60-69	0	[0.00-0.00]	0.48	[0.43-0.54]	0.52	[0.46-0.57]	0

Notes: [a]*P* value from two-sided Z test. Tests whether estimates of A are statistically different when comparing the youngest age group to older age groups. *Difference statistically significant at $P<0.05$.

Table S13. Results from ACE models. Share of variance in (type of) library borrowing attributable to shared genes (A), shared environments (C), and unique environments (E). Separate models by age group and format.

	Age	A	A 95% conf	C	C 95% conf	E	E 95% conf	Z test (*P* value)[a]
Any adult book	18-29	0.58	[0.53- 0.63]	0	[0.00- 0.00]	0.42	[0.37- 0.47]	
	30-39	0.54	[0.47- 0.61]	0	[0.00- 0.00]	0.46	[0.39- 0.53]	0.36
	40-49	0.53	[0.47- 0.60]	0	[0.00- 0.00]	0.47	[0.40- 0.53]	0.23
	50-59	0.58	[0.52- 0.65]	0	[0.00- 0.00]	0.42	[0.35- 0.48]	1
	60-69	0.59	[0.50- 0.69]	0	[0.00- 0.00]	0.41	[0.31- 0.50]	0.85
<u>Type and format</u>								
Physical books	18-29	0.60	[0.54-0.65]	0	[0.00-0.00]	0.40	[0.35-0.46]	
	30-39	0.47	[0.38-0.56]	0	[0.00-0.00]	0.53	[0.44-0.62]	0.01*
	40-49	0.55	[0.47- 0.63]	0	[0.00- 0.00]	0.45	[0.37- 0.53]	0.30
	50-59	0.60	[0.52-0.68]	0	[0.00-0.00]	0.40	[0.32-0.48]	1
	60-69	0.63	[0.53-0.74]	0	[0.00-0.00]	0.37	[0.26-0.47]	0.61
E-books	18-29	0.56	[0.48-0.64]	0	[0.00-0.00]	0.44	[0.36-0.52]	
	30-39	0.42	[0.31-0.54]	0	[0.00-0.00]	0.58	[0.46-0.69]	0.05
	40-49	0.44	[0.34-0.55]	0	[0.00-0.00]	0.56	[0.45-0.66]	0.07
	50-59	0.51	[0.40-0.62]	0	[0.00-0.00]	0.49	[0.38-0.60]	0.47
	60-69	0.53	[0.37-0.69]	0	[0.00-0.00]	0.47	[0.31-0.63]	0.74
E-audiobooks	18-29	0.61	[0.53-0.68]	0	[0.00-0.00]	0.39	[0.32-0.47]	
	30-39	0.50	[0.40-0.61]	0	[0.00-0.00]	0.5	[0.39-0.60]	0.11
	40-49	0.52	[0.42-0.61]	0	[0.00-0.00]	0.48	[0.39-0.58]	0.15
	50-59	0.62	[0.52-0.71]	0	[0.00-0.00]	0.38	[0.29-0.48]	0.88
	60-69	0.50	[0.35-0.66]	0	[0.00-0.00]	0.50	[0.34-0.65]	0.21
Fiction	18-29	0.58	[0.52-0.65]	0	[0.00-0.00]	0.42	[0.35-0.48]	
	30-39	0.53	[0.43-0.62]	0	[0.00-0.00]	0.47	[0.38-0.57]	0.40
	40-49	0.54	[0.46- 0.62]	0	[0.00- 0.00]	0.46	[0.38- 0.54]	0.44
	50-59	0.62	[0.55-0.70]	0	[0.00-0.00]	0.38	[0.30-0.45]	0.44
	60-69	0.61	[0.51- 0.71]	0	[0.00- 0.00]	0.39	[0.29- 0.49]	0.62
Non-fiction	18-29	0.56	[0.51- 0.61]	0	[0.00- 0.00]	0.44	[0.39- 0.49]	
	30-39	0.52	[0.44- 0.59]	0	[0.00- 0.00]	0.48	[0.41- 0.56]	0.40
	40-49	0.53	[0.46- 0.60]	0	[0.00- 0.00]	0.47	[0.40- 0.54]	0.51
	50-59	0.55	[0.47-0.63]	0	[0.00-0.00]	0.45	[0.37-0.53]	0.83
	60-69	0.62	[0.52-0.72]	0	[0.00-0.00]	0.38	[0.28-0.48]	0.30

Notes: [a]*P* value from two-sided Z test. Tests whether estimates of A are statistically different when comparing the youngest age group to older age groups. *Difference statistically significant at $P<0.05$.

Table S14a. Results from ACE models. Share of variance in (type of) library borrowing attributable to shared genes (A), shared environments (C), and unique environments (E). Separate models by level of education.

	Level	A	A 95% conf	C	C 95% conf	E	E 95% conf	Z test (*P* value)[a]
Any adult book	0-14 y.	0.63	[0.59- 0.67]	0	[0.00- 0.00]	0.37	[0.33- 0.41]	
	15 y.	0.50	[0.46- 0.54]	0	[0.00- 0.00]	0.5	[0.46- 0.54]	0.0000*
<u>Top 5 fiction</u>								
Crime	0-14 y.	0.65	[0.55-0.75]	0	[0.00-0.00]	0.35	[0.25-0.45]	
	15 y.	0.48	[0.40-0.55]	0	[0.00-0.00]	0.52	[0.45-0.60]	0.01*
Biographical novels	0-14 y.	0.63	[0.47-0.79]	0	[0.00-0.00]	0.37	[0.21-0.53]	
	15 y.	0.59	[0.50-0.68]	0	[0.00-0.00]	0.41	[0.32-0.50]	0.67
Thriller	0-14 y.	0.56	[0.43-0.68]	0	[0.00-0.00]	0.44	[0.32-0.57]	
	15 y.	0.52	[0.43-0.60]	0	[0.00-0.00]	0.48	[0.40-0.57]	0.60
Historical novels	0-14 y.	0.67	[0.53-0.81]	0	[0.00-0.00]	0.33	[0.19-0.47]	
	15 y.	0.60	[0.52-0.69]	0	[0.00-0.00]	0.4	[0.31-0.48]	0.40
Family novels	0-14 y.	0.60	[0.47-0.73]	0	[0.00-0.00]	0.4	[0.27-0.53]	
	15 y.	0.63	[0.54-0.72]	0	[0.00-0.00]	0.37	[0.28-0.46]	0.71
<u>Highbrow fiction</u>								
Experimental novels	0-14 y.	0.22	[-1.16-1.61]	0.49	[-0.22-1.19]	0.29	[-0.40-0.97]	
	15 y.	0.32	[-0.77-1.40]	0.19	[-0.37-0.75]	0.49	[-0.04-1.02]	0.91
Developmental novels	0-14 y.	0.57	[-0.72-1.86]	0.09	[-0.80-0.99]	0.34	[-0.09-0.77]	
	15 y.	0.61	[0.50-0.72]	0	[0.00-0.00]	0.39	[0.28-0.50]	0.95
Descriptions of societies	0-14 y.	0.37	[0.18- 0.56]	0	[0.00- 0.00]	0.63	[0.44- 0.82]	
	15 y.	0.54	[0.42-0.66]	0	[0.00-0.00]	0.46	[0.34-0.58]	0.14

Notes: [a]*P* value from two-sided Z test. Tests whether estimates of A are statistically different when comparing individuals with low education (0-14y) and high education (15 y.). *Difference statistically significant at $P<0.05$. [#]Difference statistically significant at $P<0.10$.

Table S14b. Results from ACE models. Share of variance in (type of) library borrowing attributable to shared genes (A), shared environments (C), and unique environments (E). Separate models by level of education.

	Level	A	A 95% conf	C	C 95% conf	E	E 95% conf	Z test (*P* value)[a]
Top 5 non-fiction								
Biographies	0-14 y.	0.58	[0.48-0.67]	0	[0.00-0.00]	0.42	[0.33-0.52]	
	15 y.	0.47	[0.40-0.54]	0	[0.00-0.00]	0.53	[0.46-0.60]	0.07
Home	0-14 y.	0.67	[0.54-0.80]	0	[0.00-0.00]	0.33	[0.20-0.46]	
	15 y.	0.54	[0.45-0.63]	0	[0.00-0.00]	0.46	[0.37-0.55]	0.10
Medicine	0-14 y.	0.58	[0.48-0.67]	0	[0.00-0.00]	0.42	[0.33-0.52]	
	15 y.	0.40	[0.33-0.48]	0	[0.00-0.00]	0.6	[0.52-0.67]	0.004*
Education	0-14 y.	0.58	[0.39-0.77]	0	[0.00-0.00]	0.42	[0.23-0.61]	
	15 y.	0.36	[0.23-0.50]	0	[0.00-0.00]	0.64	[0.50-0.77]	0.07
English literature	0-14 y.	0.84	[0.69-0.99]	0	[0.00-0.00]	0.16	[0.01-0.31]	
	15 y.	0.64	[0.48-0.80]	0	[0.00-0.00]	0.36	[0.20-0.52]	0.07
Type and format								
Physical books	0-14 y.	0.64	[0.58-0.69]	0	[0.00-0.00]	0.36	[0.31-0.42]	
	15 y.	0.51	[0.47- 0.55]	0	[0.00- 0.00]	0.49	[0.45- 0.53]	0.0002*
E-books	0-14 y.	0.58	[0.50-0.65]	0	[0.00-0.00]	0.42	[0.35-0.50]	
	15 y.	0.44	[0.38-0.50]	0	[0.00-0.00]	0.56	[0.50-0.62]	0.004*
E-audiobooks	0-14 y.	0.63	[0.56-0.70]	0	[0.00-0.00]	0.37	[0.30-0.44]	
	15 y.	0.51	[0.46-0.57]	0	[0.00-0.00]	0.49	[0.43-0.54]	0.01*
Fiction	0-14 y.	0.63	[0.57-0.69]	0	[0.00-0.00]	0.37	[0.31-0.43]	
	15 y.	0.54	[0.50-0.59]	0	[0.00-0.00]	0.46	[0.41-0.50]	0.02*
Non-fiction	0-14 y.	0.63	[0.58-0.68]	0	[0.00-0.00]	0.37	[0.32-0.42]	
	15 y.	0.48	[0.43- 0.52]	0	[0.00- 0.00]	0.52	[0.48- 0.57]	0.0000*

Notes: [a]*P* value from two-sided Z test. Tests whether estimates of A are statistically different when comparing individuals with low education (0-14y) and high education (15 y.). *Difference statistically significant at $P<0.05$. [#]Difference statistically significant at $P<0.10$.

Table S15a. Results from ACE models. Share of variance in (type of) library borrowing attributable to shared genes (A), shared environments (C), and unique environments (E). Separate models by level of income.

	Level	A	A 95% conf	C	C 95% conf	E	E 95% conf	Z test (*P* value)[a]
Any adult book	Below median	0.62	[0.58- 0.67]	0	[0.00- 0.00]	0.38	[0.33- 0.42]	
	Above median	0.53	[0.49- 0.57]	0	[0.00- 0.00]	0.47	[0.43- 0.51]	0.002*
<u>Top 5 fiction</u>								
Crime	Below median	0.64	[0.51-0.76]	0	[0.00-0.00]	0.36	[0.24-0.49]	
	Above median	0.49	[0.41-0.56]	0	[0.00-0.00]	0.51	[0.44-0.59]	0.05
Biographical novels	Below median	0.81	[0.68-0.93]	0	[0.00-0.00]	0.19	[0.07-0.32]	
	Above median	0.54	[0.45-0.64]	0	[0.00-0.00]	0.46	[0.36-0.55]	0.001*
Thriller	Below median	0.61	[0.46-0.75]	0	[0.00-0.00]	0.39	[0.25-0.54]	
	Above median	0.50	[0.42-0.58]	0	[0.00-0.00]	0.5	[0.42-0.58]	0.19
Historical novels	Below median	0.58	[-0.33-1.49]	0.17	[-0.47-0.80]	0.25	[-0.04-0.55]	
	Above median	0.57	[0.48-0.66]	0	[0.00-0.00]	0.43	[0.34-0.52]	0.98
Family novels	Below median	0.72	[0.56-0.88]	0	[0.00-0.00]	0.28	[0.12-0.44]	
	Above median	0.63	[0.54-0.72]	0	[0.00-0.00]	0.37	[0.28-0.46]	0.34
<u>Highbrow fiction</u>								
Experimental novels	Below median	0.84	[0.73-0.95]	0	[0.00-0.00]	0.16	[0.05-0.27]	
	Above median	0	[0.00-0.00]	0.42	[0.38-0.47]	0.58	[0.53-0.62]	0
Developmental novels	Below median	0.78	[0.61-0.95]	0	[0.00-0.00]	0.22	[0.05-0.39]	
	Above median	0.60	[0.49-0.71]	0	[0.00-0.00]	0.4	[0.29-0.51]	0.08
Descriptions of societies	Below median	0.57	[0.36-0.79]	0	[0.00-0.00]	0.43	[0.21-0.64]	
	Above median	0.54	[0.42-0.65]	0	[0.00-0.00]	0.46	[0.35-0.58]	0.81

Notes: [a]*P* value from two-sided Z test. Tests whether estimates of A are statistically different when comparing individuals whose income is below and above the median. *Difference statistically significant at $P<0.05$.

Table S15b. Results from ACE models. Share of variance in (type of) library borrowing attributable to shared genes (A), shared environments (C), and unique environments (E). Separate models by level of income.

	Level	A	A 95% conf	C	C 95% conf	E	E 95% conf	Z test (*P* value)[a]
<u>Top 5 non-fiction</u>								
Biographies	Below median	0.62	[0.53-0.71]	0	[0.00-0.00]	0.38	[0.29-0.47]	
	Above median	0.46	[0.39-0.53]	0	[0.00-0.00]	0.54	[0.47-0.61]	0.01*
Home	Below median	0.51	[0.36-0.66]	0	[0.00-0.00]	0.49	[0.34-0.64]	
	Above median	0.62	[0.54-0.70]	0	[0.00-0.00]	0.38	[0.30-0.46]	0.20
Medicine	Below median	0.57	[0.48-0.66]	0	[0.00-0.00]	0.43	[0.34-0.52]	
	Above median	0.42	[0.34-0.50]	0	[0.00-0.00]	0.58	[0.50-0.66]	0.01*
Education	Below median	0.56	[0.40-0.72]	0	[0.00-0.00]	0.44	[0.28-0.60]	
	Above median	0.36	[0.21-0.51]	0	[0.00-0.00]	0.64	[0.49-0.79]	0.08
English literature	Below median	0.85	[0.72-0.98]	0	[0.00-0.00]	0.15	[0.02-0.28]	
	Above median	0.39	[0.21-0.56]	0	[0.00-0.00]	0.61	[0.44-0.79]	0.0000*
<u>Type and format</u>								
Physical books	Below median	0.62	[0.57-0.67]	0	[0.00-0.00]	0.38	[0.33-0.43]	
	Above median	0.54	[0.50-0.59]	0	[0.00-0.00]	0.46	[0.41-0.50]	0.02*
E-books	Below median	0.58	[0.51-0.66]	0	[0.00-0.00]	0.42	[0.34-0.49]	
	Above median	0.45	[0.39-0.51]	0	[0.00-0.00]	0.55	[0.49-0.61]	0.01*
E-audiobooks	Below median	0.66	[0.59-0.73]	0	[0.00-0.00]	0.34	[0.27-0.41]	
	Above median	0.51	[0.45-0.57]	0	[0.00-0.00]	0.49	[0.43-0.55]	0.001*
Fiction	Below median	0.64	[0.58-0.70]	0	[0.00-0.00]	0.36	[0.30-0.42]	
	Above median	0.56	[0.51-0.60]	0	[0.00-0.00]	0.44	[0.40-0.49]	0.03*
Non-fiction	Below median	0.60	[0.56- 0.65]	0	[0.00- 0.00]	0.4	[0.35- 0.44]	
	Above median	0.52	[0.48- 0.56]	0	[0.00- 0.00]	0.48	[0.44- 0.52]	0.01*

Notes: [a]*P* value from two-sided Z test. Tests whether estimates of A are statistically different when comparing individuals whose income is below and above the median. *Difference statistically significant at $P<0.05$.

Table S16. Share and count of borrowers across full population, females and males, and loan genre/types.

	Share			N borrowers		
	Full pop	Female	Male	Full pop	Female	Male
Any adult book	0.17	0.25	0.09	11,543	8,370	3,098
Top 5 fiction						
Crime	0.04	0.05	0.02	2,716	1,674	688
Biographical novels	0.02	0.03	0.01	1,358	1,004	344
Thriller	0.03	0.04	0.02	2,037	1,339	688
Historical novels	0.02	0.03	0.01	1,358	1,004	344
Family novels	0.02	0.03	0.01	1,358	1,004	344
Highbrow fiction						
Experimental novels	0	0	0	136	100	34
Developmental novels	0.01	0.02	0	679	670	138
Descriptions of societies	0.01	0.02	0.01	679	670	344
Top 5 non-fiction						
Biographies	0.04	0.07	0.02	2,716	2,344	688
Home	0.02	0.04	0.01	1,358	1,339	344
Medicine	0.04	0.07	0.02	2,716	2,344	688
Education	0.01	0.02	0.01	679	670	344
English literature	0.01	0.01	0.01	679	335	172
Type and format						
Physical books	0.11	0.17	0.06	7,469	5,692	2,065
E-books	0.06	0.09	0.03	4,074	3,013	1,033
E-audiobooks	0.06	0.09	0.04	4,074	3,013	1,377
Fiction	0.10	0.15	0.05	6,790	5,022	1,721
Non-fiction	0.14	0.20	0.08	9,506	6,696	2,754

Table S17. Share and count of borrowers across education and income, and loan genre/types.

	Share				N borrowers			
	High edu	Low edu	High inc	Low inc	High edu	Low edu	High inc	Low inc
Any adult book	0.23	0.13	0.19	0.16	5,965	5,400	5,621	6,130
Top 5 fiction								
Crime	0.05	0.03	0.05	0.02	1,297	1,246	1,479	766
Biographical novels	0.03	0.01	0.03	0.01	778	415	888	383
Thriller	0.04	0.02	0.04	0.02	1,037	831	1,183	766
Historical novels	0.03	0.01	0.03	0.01	778	415	888	383
Family novels	0.03	0.01	0.03	0.01	778	415	888	383
Highbrow fiction								
Experimental novels	0.00	0.00	0.00	0.00	104	42	59	77
Developmental novels	0.02	0.01	0.02	0.01	519	415	592	383
Descriptions of societies	0.02	0.01	0.02	0.01	519	415	592	383
Top 5 non-fiction								
Biographies	0.06	0.03	0.05	0.04	1,556	1,246	1,479	1,533
Home	0.04	0.01	0.03	0.02	1,037	415	888	766
Medicine	0.06	0.03	0.05	0.04	1,556	1,246	1,479	1,533
Education	0.02	0.01	0.02	0.01	519	415	592	383
English literature	0.01	0.01	0.01	0.01	259	415	296	383
Type and format								
Physical books	0.16	0.09	0.12	0.11	4,149	3,738	3,550	4,215
E-books	0.09	0.05	0.07	0.06	2,334	2,077	2,071	2,299
E-audiobooks	0.09	0.05	0.08	0.05	2,334	2,077	2,367	1,916
Fiction	0.14	0.08	0.12	0.08	3,631	3,323	3,550	3,065
Non-fiction	0.19	0.11	0.15	0.13	4,927	4,569	4,438	4,981

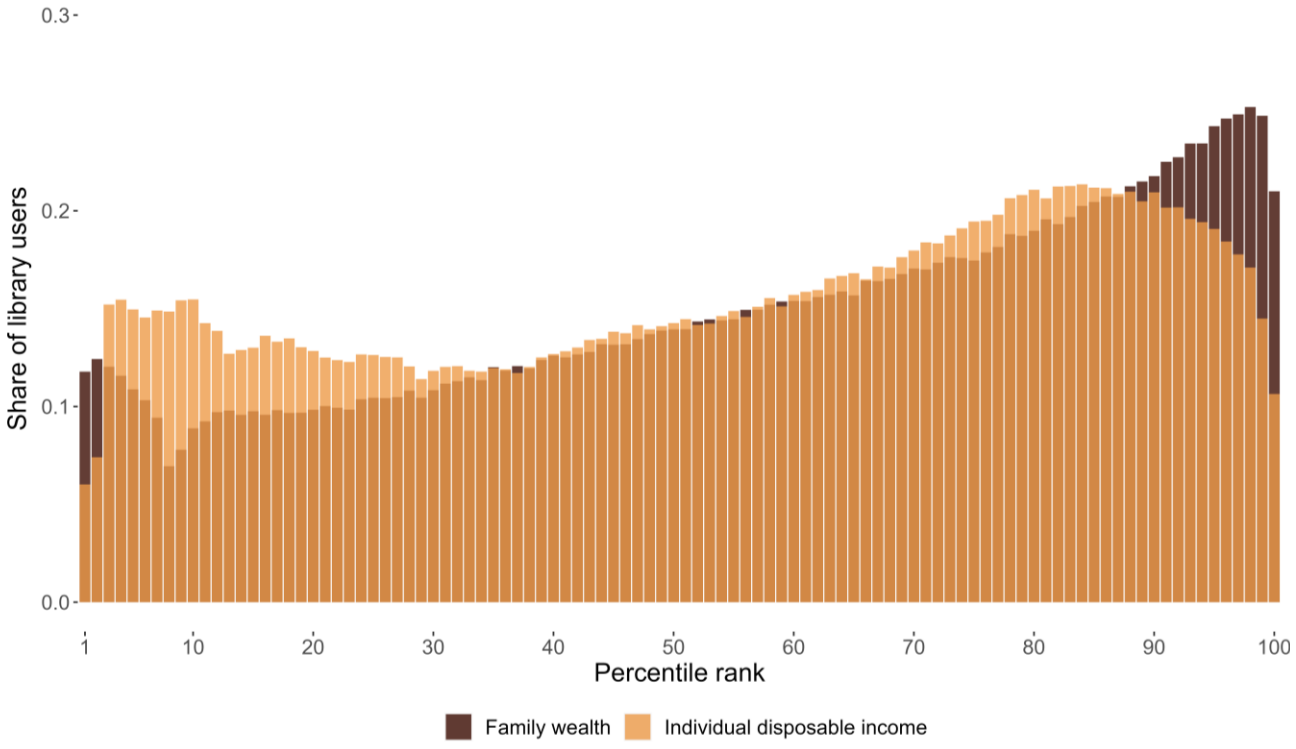

Figure S1. Share of individuals that has taken out at least one book from the adult book collection in 2021, by family disposable income and wealth percentile rank. The figure shows that the likelihood of taking out at least one book from the library increases with family disposable income and wealth, but also that differences in library use are not very large.
